# E-health literacy status and its influencing factors among college students majoring in sports

**DOI:** 10.3389/fpubh.2026.1931785

**Published:** 2026-09-03

**Authors:** Yue Yin, Jing Chen, Wenqing Zhao, Linwang Hu, Hang Cai, Liang Gao

**Affiliations:** 1 School of Physical Education, Lianyungang Normal University, Lianyungang, Jiangsu, China; 2 School of Sport Science, Nanjing Normal University, Nanjing, Jiangsu, China; 3 School of Physical Education, Nanjing Xiaozhuang University, Nanjing, Jiangsu, China; 4 School of Physical Education, Huaiyin Normal University, Huaian, Jiangsu, China; 5 Jiangsu Gymnastics Sports Association, Nanjing, Jiangsu, China; 6 Nanjing Sport Institute, Nanjing, Jiangsu, China

**Keywords:** e-health literacy, influencing factors, regression analysis, sport majors, university students

## Abstract

**Objective:**

To understand the level of e-health literacy among sports majors and its influencing factors, providing reference for developing measures to improve e-health literacy in this population.

**Methods:**

An online survey was conducted among college students from 6 universities in Jiangsu Province using an e-health literacy questionnaire. SPSS 26.0 statistical software was used for χ^2^ test. *t*-test, one-way ANOVA, and multiple linear regression analysis were employed to explore the influencing factors of e-health literacy level.

**Results:**

Among the 923 respondents, 722 (78.2%) were male and 201 (21.8%) were female. The average e-health literacy score was 41.91 ± 6.90, with an adequate rate of 22.2% (205 individuals). Influencing factor analysis showed that ethnicity (95%CI: −3.641 ~ −0.408), gender (95%CI: 1.292 ~ 3.445), self-rated health status compared with peers (95%CI: −1.276 ~ −0.100), use of smart wearable devices (95%CI: 0.100 ~ 0.794), harmony with surrounding people (95%CI: 0.379 ~ 1.733), frequency of searching health knowledge on the Internet (95%CI: 0.246 ~ 1.099), computer proficiency (95%CI: 0.230 ~ 1.569), days waking up feeling energetic in the morning (95%CI: 0.188 ~ 1.207), frequency of consuming fish, poultry, meat and eggs (95%CI: 0.194 ~ 1.331), and types of health knowledge content searched (95%CI: 0.023 ~ 1.556) were significant factors affecting e-health literacy among college students.

**Conclusion:**

The e-health literacy level of sports majors is relatively low, with associated factors spanning multiple dimensions. Targeted interventions should prioritize male students, those with poorer self-rated health, and those with weaker interpersonal relationships. Strengthening digital skills and active information practice is a key strategy for improving e-health literacy in this population.

## Introduction

With the rapid development of Internet technology, digital health information has become the primary channel for the public to access health knowledge. E-health literacy refers to the comprehensive capacity of individuals to access, understand, and appraise health information from electronic resources, and to apply the obtained information to address health problems and facilitate health related decision making (1), encompassing three progressive dimensions: functional (basic reading and comprehension skills), interactive (active extraction and application of information), and critical (critical analysis and utilization of health information) (2). The present study is guided by Sørensen et al.’s (2) integrative model of health literacy, which conceptualizes health literacy as competencies to access, understand, appraise, and apply health information, and identifies demographic, social, and personal factors as its antecedents. Following this framework, we selected demographic, health related, psychosocial, and digital proficiency variables to examine their associations with e-health literacy among sport majors.

University students, as digital natives, constitute the predominant users of Internet based health information. Empirical studies have revealed marked regional and demographic disparities in e-health literacy levels among Chinese university students. Specifically, the proficiency rate was reported at 7.91% among non-medical students in Wuhan (3), 16.6% among university students in Henan Province (4), 20.3% among university students in Guangzhou (5), and 22.97% among university students in Nanjing (6), collectively indicating a generally suboptimal status quo of e-health literacy in this population. This pattern is not unique to China. A recent cross national comparative study of university students in Japan, the United States, and India also reported suboptimal digital health literacy levels, with mean scores ranging from 2.89 to 3.10 on a 4-point scale (7), suggesting that inadequate e-health literacy among young adults represents a shared international concern rather than a country-specific phenomenon.

Notably, however, prior research specifically targeting e-health literacy among sport majors remains conspicuously scarce. While recent international studies have begun to examine e-health literacy in physically active populations, for instance, Geiger et al. (8) investigated 373 German athletes and found significant associations with injury frequency and substance use—sport majors, who are neither elite athletes nor general students, remain largely overlooked in both domestic and international research.

This gap is particularly concerning given that sport majors are not merely a subgroup of university students, they represent a distinct population with unique characteristics. Unlike their peers in other disciplines, they undergo a training intensive curriculum that places heightened demands on physical performance and injury prevention, which may intensify their need for timely and reliable health information. Concurrently, their academic and training schedules often limit opportunities for conventional health education, making digital health resources particularly salient for this group. Furthermore, their health information seeking behaviors may differ from those of general students due to the predominance of coach mediated communication and hands on learning in their educational environment. These distinctive features suggest that findings derived from general student populations are unlikely to be directly transferable to sport majors. As prospective physical educators, these students bear the social responsibility of teaching and nurturing future generations and disseminating scientific fitness knowledge to the general public (9). Moreover, preliminary evidence indicates that general health literacy among sport majors remains critically low, a recent large sample study reported an overall proficiency rate of only 31.49% among this population, with basic health skills and medical care literacy falling below 30% (10), raising the question of whether their digital specific health literacy, i.e., e-health literacy, may face similar or even greater challenges. Additionally, qualitative findings suggest that sport majors exhibit a practical oriented and curious approach to using digital health tools (11), making them an especially suitable target population for e-health literacy research.

Given this confluence of population distinctiveness, professional salience, and empirical indication of potential vulnerability, a focused investigation into the e-health literacy of sport majors is both academically warranted and practically urgent. Accordingly, the present study aims to investigate the current status of their e-health literacy and analyze its influencing factors, thereby providing empirical evidence for health education reform in physical education institutions and the cultivation of professionally competent sport graduates. The selection of variables demographic, health related, psychosocial, and digital proficiency factors was guided by the eHealth Literacy Model (2) and prior empirical evidence.

The findings are expected to provide empirical evidence for health education reform in physical education institutions and inform the development of targeted interventions to improve digital health competencies in this population.

### Research questions

1. What is the current level of e-health literacy among sport majors in Chinese universities?
2. What demographic, health related, psychosocial, and digital proficiency factors are significantly associated with e-health literacy in this population?
3. Which of these factors demonstrate the strongest associations with e-health literacy outcomes?

## Methods

### Sampling and participants

A stratified random cluster sampling method was employed. From October to December 2025, six undergraduate institutions were selected from three administrative regions of Jiangsu Province (northern, central, and southern Jiangsu), with two institutions from each region. Jiangsu Province was divided into three administrative regions—northern, central, and southern Jiangsu—based on geographical location and socioeconomic development levels. Southern Jiangsu (e.g., Suzhou, Wuxi, Changzhou) is the most economically developed region with relatively abundant educational and healthcare resources; central Jiangsu (e.g., Nanjing, Zhenjiang, Yangzhou) represents a transitional zone with moderate development levels; and northern Jiangsu (e.g., Xuzhou, Lianyungang, Huai’an) is characterized by relatively lower economic development and fewer health education resources. This regional stratification was intended to capture the diversity of digital health infrastructure and educational contexts across the province. All institutions offer full-time undergraduate programs in physical education and sport science. Within the selected universities, one to two classes were chosen from each academic year (Years 1–4) and major orientation (Physical Education, Social Sports, Athletic Training, etc.). All students in the selected classes were invited to participate in this survey. Questionnaires were distributed after participants were fully informed of the study purpose, completion instructions, and provided written consent.

To determine the sample size, the formula for estimating the mean $n=\frac{\sigma^{2}\cdot Z_{1-\frac{\alpha }{2}}^{2}}{\Delta^{2}}$ was used. When α set at 0.05, $Z_{1-\frac{\alpha }{2}}^{2}=1.96^{2}$ , the margin of error from the preliminary studies was Δ = 1.59. After substituting these values into the formula, the sample size for this study was calculated to be 181 participants. The total sample size for the six schools was 1,086. Since the number of students in each class is not uniform, bringing the total sample size to 1,101 participants. After excluding those with erroneous, incomplete, or evidently random responses, 923 valid questionnaires were retained, yielding an effective response rate of 83.8%. This study was reviewed and approved by the Science and Technology Industry Office of Lianyungang Normal University.

### E-health literacy questionnaire

This study utilized the e-Health Literacy Scale (EHLS), developed by Chiang et al. (12, 13) from Taiwan, China. The applicability, reliability, and validity of this instrument have been substantiated in research by Zhang et al. (14). The questionnaire comprises four sections: demographic characteristics, health behavior survey, e-health utilization survey, and e-health literacy assessment. The EHLS consists of three dimensions—functional e-health literacy (Items 1–3), interactive e-health literacy (Items 4–7), and critical e-health literacy (Items 8–12)—encompassing 12 items in total. A 5 point Likert scale was employed for scoring. Notably, the three items in Dimension 1 were reverse scored (5 = strongly disagree to 1 = strongly agree), whereas the nine items in Dimensions 2 and 3 were scored directly (1 = strongly disagree to 5 = strongly agree). The total possible score ranges from 12 to 60, with higher scores indicating superior e-health literacy.

### Operational definitions and conceptual framework

*E-health literacy proficiency*: following established criteria (15), e-health literacy proficiency was operationalized as achieving a score of ≥48 (≥80% of the maximum possible score of 60). The prevalence of e-health literacy was calculated as the proportion of participants meeting this proficiency threshold relative to the total sample.

*Functional e-health literacy*: refers to foundational competencies in reading, writing, and numeracy within digital health contexts, encompassing basic health concept comprehension. Among university students, typical information seeking behaviors include: acquiring knowledge about infectious diseases (e.g., HIV/AIDS awareness), accessing emergency management protocols for common sports injuries, and retrieving health policy updates or lecture announcements.

*Interactive e-health literacy*: denotes the capacity for active engagement, interpersonal interaction, and knowledge application in digital environments—representing a higher order competency than functional literacy. Manifestations among university students include: digital patient provider interactions (e.g., online medical consultations), participation in health focused online communities (e.g., initiating discussions or sharing experiences in health forums), knowledge to behavior translation (e.g., implementing personalized exercise prescriptions), and advanced engagement with intelligent tools (e.g., customizing settings in health related mobile applications).

*Critical e-health literacy*: refers to the capability to analyze, evaluate, and respond to online health information (4). Among university students, this is evidenced by: identifying misinformation, pseudoscientific claims, and statistical fallacies (e.g., conflating correlation with causation) on social media; recognizing social correlates of health (e.g., mechanisms linking academic pressure to wellbeing); and leveraging digital platforms to advocate for campus health policy improvements (e.g., promoting nutritional enhancements in dining facilities), thereby safeguarding individual and collective health rights.

### Statistical analysis

Statistical analyses were conducted using SPSS 26.0 (IBM Corp., Armonk, NY, USA). Categorical variables were described as frequencies or percentages (%), with between group comparisons performed using the chi-square (χ^2^) test. Continuous variables were presented as mean ± standard deviation (x̄ ± s), and between group comparisons were conducted using independent samples *t*-tests (for two groups) or one-way analysis of variance (ANOVA) (for multiple groups). Statistical significance was set at *p* < 0.05. Multiple linear regression analysis was employed to identify factors influencing e-health literacy levels. Before conducting the regression analysis, the normality of the e-health literacy total score was assessed using the Shapiro–Wilk test and visual inspection of Q-Q plots. Although the Shapiro–Wilk test indicated a significant deviation from normality (*p* < 0.001), parametric tests were considered appropriate given the large sample size (*n* = 923), as they are robust to violations of normality in large samples. Homogeneity of variance was evaluated using Levene‘s test. The significance level (*α*) was set at 0.05 for all tests.

## Results

### Demographic characteristics

A total of 923 sport majors participated in this study. The demographic characteristics of the sample are presented in Table 1. The majority of participants were male (78.2%), of Han ethnicity (92.8%), and from rural areas (67.0%). Participants were distributed across all four academic years, with the largest proportion being third-year students (31.5%).

**Table 1:** Demographic characteristics of the sample (*n* = 923).

Variable	Categories	*n* (%)
Household registration	Urban	305 (33.0)
	Rural	618 (67.0)
Ethnicity	Han	857 (92.8)
	Minority	66 (7.2)
Gender	Male	722 (78.2)
	Female	201 (21.8)
Academic year	1	275 (29.8)
	2	285 (30.9)
	3	291 (31.5)
	4	72 (7.8)

### Scores of e-health literacy dimensions among sport majors

Among the 923 sport majors surveyed, 205 demonstrated proficient e-health literacy, yielding a prevalence of 22.2%. The observed scores ranged from 17 to 60, with a mean of 41.91 ± 6.90. Dimension specific scores were as follows: functional e-health literacy, 9.61 ± 2.57; critical e-health literacy, 17.78 ± 3.73; and interactive e-health literacy, 14.52 ± 2.99.

### Statistical analysis of e-health literacy among sport majors

Independent samples *t*-tests and one way ANOVA were conducted to examine differences in e-health literacy across demographic and behavioral characteristics. Statistically significant differences were identified for 25 factors, including household registration (hukou), ethnicity, gender, self-rated academic performance, and confidence in retrieving valid health information online (*p* < 0.05).

Effect size analysis further revealed that five factors demonstrated moderate practical significance: ethnicity (*d* = 0.46), gender (*d* = −0.35), smoking status (*d* = −0.33), perceived utility of Internet health information for health decision making (*d* = −0.55), and confidence in retrieving valid health information online (*d* = −0.54). Detailed results are presented in Table 2.

**Table 2:** Comparison of e-health literacy scores by demographic characteristics (*n* = 923).

Variable	Categories	*n* (%)	*x̄* ± s	T/F	*p*	Effect size
Household registration	Urban	305 (33.0)	42.57 ± 6.8	2.06	0.040	0.14
	Rural	618 (67.0)	41.58 ± 6.9			
Ethnicity	Han	857 (92.8)	42.13 ± 7.0	4.586	<0.001	0.46
	Minority	66 (7.2)	39.00 ± 5.2			
Gender	Male	722 (78.2)	41.39 ± 6.9	−4.314	<0.001	−0.35
	Female	201 (21.8)	43.75 ± 6.7			
Academic year	1	275 (29.8)	41.91 ± 6.7	4.855	0.002	0.02
	2	285 (30.9)	42.40 ± 6.8			
	3	291 (31.5)	40.90 ± 6.8			
	4	72 (7.8)	44.01 ± 7.7			
Weekly exercise frequency	Never	8 (0.9)	36.88 ± 5.8	4.092	0.003	0.02
	Daily	432 (46.8)	42.47 ± 7.0			
	4–6times per week	294 (31.9)	42.11 ± 7.0			
	2–3times per week	170 (18.4)	40.68 ± 6.4			
	Once per week	19 (2.1)	39.00 ± 5.8			
Exercise duration	Never	8 (0.9)	36.88 ± 5.8	2.012	0.111	0.01
	>60 min	672 (72.8)	42.11 ± 7.1			
	30–60 min	224 (24.3)	41.60 ± 6.1			
	<30 min	19 (2.1)	40.53 ± 7.3			
Use of smart wearable devices	Never use	318 (34.5)	40.70 ± 6.7	3.774	0.005	0.02
	≥1 year	357 (38.7)	42.61 ± 7.1			
	6–12 months	83 (9.0)	42.25 ± 6.2			
	3–6 months	66 (7.2)	42.58 ± 7.8			
	Less than 3 months	99 (10.7)	42.51 ± 6.4			
Self-rated health status	Excellent	588 (63.7)	42.52 ± 7.0	8.172	<0.001	0.03
	Good	226 (24.5)	41.68 ± 6.5			
	Fair	101 (10.9)	38.92 ± 6.2			
	Poor	8 (0.9)	41.00 ± 8.1			
Self-rated academic performance	Excellent	210 (22.8)	43.87 ± 7.8	9.868	<0.001	0.03
	Good	376 (40.7)	41.95 ± 6.7			
	Average	312 (33.8)	40.68 ± 6.1			
	Pass or below	25 (2.7)	40.00 ± 7.9			
Father’s education level	Primary school or below	135 (14.6)	40.52 ± 6.2	3.69	0.012	0.01
	Junior high school	356 (38.6)	41.79 ± 7.1			
	Senior high school	284 (30.8)	42.02 ± 6.9			
	Bachelor’s degree or above	148 (16.0)	43.22 ± 6.8			
Mother’s education level	Primary school or below	202 (21.9)	41.32 ± 6.3	1.042	0.373	0.00
	Junior high school	370 (40.1)	41.79 ± 6.7			
	Senior high school	224 (24.3)	42.37 ± 7.5			
	Bachelor’s degree or above	127 (13.8)	42.36 ± 7.3			
Daily sleep duration	<7 h	212 (23.0)	41.59 ± 7.1	0.352	0.703	0.00
	7–8 h	577 (62.5)	42.05 ± 6.7			
	>8 h	134 (14.5)	41.80 ± 7.5			
Days feeling energetic per week	0 day	109 (11.8)	39.06 ± 7.2	12.198	<0.001	0.04
	1–2 days	303 (32.8)	41.27 ± 6.2			
	3–5 days	362 (39.2)	42.52 ± 6.8			
	>5 days	149 (16.1)	43.81 ± 7.6			
Days consuming vegetables per week	Never	14 (1.5)	35.86 ± 7.4	7.184	<0.001	0.02
	1–2 days	102 (11.1)	41.34 ± 6.3			
	3–5 days	224 (24.3)	40.90 ± 6.1			
	Everyday	583 (63.2)	42.54 ± 7.2			
Days consuming fruits per week	Never	48 (5.2)	39.25 ± 6.6	8.074	<0.001	0.03
	1–2 days	406 (44.0)	41.15 ± 6.5			
	3–5 days	267 (28.9)	42.34 ± 7.0			
	Everyday	202 (21.9)	43.49 ± 7.3			
Days consuming fish, poultry, meat, and eggs per week	Never	12 (1.3)	39.58 ± 8.8	11.046	<0.001	0.03
	1–2 days	176 (19.1)	39.65 ± 5.9			
	3–5 days	241 (26.1)	41.54 ± 6.3			
	Everyday	494 (53.5)	42.95 ± 7.3			
Days consuming milk or dairy products per week	Never	47 (5.1)	39.06 ± 7.4	12.929	<0.001	0.04
	1–2 days	324 (35.1)	40.47 ± 6.3			
	3–5 days	271 (29.4)	42.63 ± 6.5			
	Everyday	281 (30.4)	43.35 ± 7.4			
Alcohol consumption frequency	Never	268 (29.0)	42.65 ± 7.4	3.662	0.006	0.02
	Rarely	346 (37.5)	42.23 ± 6.8			
	Seldom	144 (15.6)	40.55 ± 6.0			
	Sometimes	144 (15.6)	41.60 ± 6.2			
	Often	21 (2.3)	38.62 ± 9.3			
Smoking status	Yes	179 (19.4)	40.08 ± 6.7	−3.981	<0.001	−0.33
	No	744 (80.6)	42.35 ± 6.9			
Interpersonal harmony	Poor	7 (0.8)	34.43 ± 6.1	16.942	<0.001	0.05
	Fair	92 (10.0)	38.39 ± 7.5			
	Good	502 (54.4)	41.68 ± 6.1			
	Excellent	322 (34.9)	43.43 ± 7.4			
Frequency of Internet health information seeking	Never	48 (5.2)	38.00 ± 7.5	13.584	<0.001	0.06
	Several times per year	170 (18.4)	40.28 ± 6.7			
	Several times per month	319 (34.6)	41.31 ± 6.3			
	Several times per week	266 (28.8)	43.36 ± 6.4			
	Several times per day	120 (13.0)	44.13 ± 8.2			
Number of channels for obtaining health knowledge online	1	49 (5.3)	37.16 ± 8.1	5.771	<0.001	0.06
	2	115 (12.5)	40.97 ± 5.5			
	3	197 (21.3)	40.85 ± 6.4			
	4	128 (13.9)	42.29 ± 6.1			
	5	149 (16.1)	43.68 ± 7.3			
	6	95 (10.3)	41.83 ± 6.2			
	7	76 (8.2)	42.16 ± 6.4			
	8	20 (2.2)	42.00 ± 7.5			
	9	17 (1.8)	41.76 ± 7.6			
	10	7 (0.8)	42.43 ± 6.6			
	≥11	70 (7.6)	45.06 ± 8.6			
Diversity of health information topics searched	1	60 (6.5)	38.60 ± 8.5	15.019	<0.001	0.08
	2	214 (23.2)	41.07 ± 6.2			
	3	251 (27.2)	40.76 ± 6.1			
	4	226 (24.5)	42.45 ± 6.5			
	5	46 (5.0)	42.50 ± 6.9			
	≥6	126 (13.7)	45.99 ± 7.6			
Number of social networks registered and used	1	46 (5.0)	39.65 ± 8.5	4.92	<0.001	0.04
	2	136 (14.7)	40.71 ± 6.8			
	3	178 (19.3)	40.99 ± 6.4			
	4	225 (24.4)	41.82 ± 6.1			
	5	110 (11.9)	41.51 ± 6.9			
	6	115 (12.5)	43.52 ± 7.0			
	7	21 (2.3)	43.33 ± 5.7			
	8	40 (4.3)	44.23 ± 7.6			
	≥9	52 (5.6)	45.46 ± 8.1			
Computer proficiency	Very poor	20 (2.2)	39.1 ± 10.0	11.42	<0.001	0.05
	Poor	73 (7.9)	39.52 ± 5.8			
	Fair	648 (70.2)	41.50 ± 6.6			
	Good	148 (16.0)	44.29 ± 6.7			
	Excellent	34 (3.7)	45.97 ± 9.0			
Number of evaluation methods used for searched health information	1	85 (9.2)	39.00 ± 7.7	6.655	<0.001	0.04
	2	251 (27.2)	41.69 ± 6.4			
	3	291 (31.5)	41.87 ± 6.9			
	4	197 (21.3)	42.11 ± 6.2			
	5	24 (2.6)	43.25 ± 7.5			
	≥6	75 (8.1)	45.08 ± 7.9			
Perceived usefulness of Internet health information for health decision-making	No	76 (8.2)	38.47 ± 6.8	−4.577	<0.001	−0.55
	Yes	847 (91.8)	42.21 ± 6.8			
Confidence in retrieving valid health information online	No	84 (9.1)	38.54 ± 7.0	−4.752	<0.001	−0.54
	Yes	839 (90.9)	42.24 ± 6.8			

Effect size: Cohen’s d for *t*-tests, η^2^ for ANOVA. *p* < 0.05 indicates statistical significance.

### Multiple linear regression analysis of factors influencing E-health literacy among sport majors

To identify independent predictors of e-health literacy, a multiple linear regression analysis was conducted with the total e-health literacy score as the dependent variable. Independent variables that exhibited statistically significant associations in prior univariate analyses (independent samples *t*-tests and one way ANOVA) were entered as predictors.

A multiple linear regression model was constructed to identify independent predictors of e-health literacy among sport majors, with the total e-health literacy score as the dependent variable. All variables that demonstrated statistically significant associations (*p* < 0.05) in the univariate analyses were simultaneously entered as independent predictors using the Enter method. This analysis identified ethnicity, gender, self-rated health status, social integration, frequency of Internet use for health information seeking, computer literacy, diversity of health information topics accessed, smart wearable device use, and days feeling energetic per week as significant associated factors. The overall regression model was statistically significant, *F*(24, 898) = 9.439, *p* < 0.001, indicating that the set of predictors collectively explained a significant proportion of the variance in e-health literacy scores. The model accounted for 20.1% of the variance (R^2^ = 0.201), with an adjusted R^2^ of 0.180. Collinearity diagnostics confirmed that all VIF values were below 10, indicating no significant multicollinearity. Detailed regression coefficients (B, SE, β, t, p, and 95% CI) for each predictor are presented in Table 3.

**Table 3:** Multiple linear regression analysis of factors influencing e-health literacy (*n* = 923).

Variable	B	SE	Beta	*t*	*p*	95%CI
Constant	25.022	3.144		7.96	<0.001	18.852 ~ 31.191
Household registration	−0.265	0.481	−0.018	−0.55	0.582	−1.208 ~ 0.679
Ethnicity	−2.025	0.824	−0.076	−2.458	0.009	−3.641 ~ −0.408
Gender	2.368	0.548	0.142	4.319	<0.001	1.292 ~ 3.445
Smoking status (including e-cigarettes)	0.626	0.574	0.036	1.091	0.276	−0.501 ~ 1.753
Perceived usefulness of Internet health information for health decision-making	1.47	1.025	0.059	1.434	0.152	−0.542 ~ 3.482
Confidence in retrieving valid health information online	0.817	0.983	0.034	0.831	0.406	−1.112 ~ 2.746
Academic year	0.01	0.232	0.001	0.041	0.967	−0.446 ~ 0.465
Weekly exercise frequency	−0.147	0.266	−0.018	−0.552	0.581	−0.668 ~ 0.375
Smart wearable device usage (e.g., fitness bands)	0.447	0.177	0.083	2.528	0.012	0.100 ~ 0.794
Self-rated health status	−0.688	0.3	−0.072	−2.295	0.022	−1.276 ~ −0.1
Self-rated academic performance	−0.258	0.285	−0.03	−0.907	0.365	−0.817 ~ 0.301
Father’s education level	−0.053	0.25	−0.007	−0.211	0.833	−0.544 ~ 0.438
Days feeling energetic per week	0.697	0.26	0.09	2.685	0.007	0.188 ~ 1.207
Days consuming vegetables per week	0.015	0.312	0.002	0.047	0.963	−0.599 ~ 0.628
Days consuming fruits per week	−0.239	0.286	−0.03	−0.835	0.404	−0.8 ~ 0.322
Days consuming fish, poultry, meat, and eggs per week	0.762	0.29	0.091	2.632	0.009	0.194 ~ 1.331
Days consuming milk or dairy products per week	0.498	0.272	0.066	1.832	0.067	−0.036 ~ 1.031
Alcohol consumption frequency	−0.24	0.202	−0.038	−1.187	0.235	−0.636 ~ 0.157
Interpersonal harmony	1.056	0.345	0.1	3.061	0.002	0.379 ~ 1.733
Frequency of Internet health information seeking	0.673	0.217	0.104	3.095	0.002	0.246 ~ 1.099
Computer proficiency	0.899	0.341	0.089	2.636	0.009	0.23 ~ 1.569
Number of channels for obtaining health knowledge online (types)	0.435	0.333	0.047	1.304	0.193	−0.219 ~ 1.089
Number of health information topics searched (types)	0.789	0.391	0.079	2.02	0.044	0.023 ~ 1.556
Number of evaluation methods used for searched health information (types)	−0.742	0.416	−0.068	−1.783	0.075	−1.558 ~ 0.075
Number of social networks registered and used (types)	0.582	0.365	0.056	1.592	0.112	−0.135 ~ 1.298

Model summary: *F*(24, 898) = 9.439, *p* < 0.001, R^2^ = 0.201, Adjusted R^2^ = 0.180.

Variable coding for significant predictors: Ethnicity (Han = 1, Minority = 2); Gender (Male = 1, Female = 2); Self-rated health (Poor = 1, Fair = 2, Good = 3, Excellent = 4); Interpersonal harmony (Poor = 1, Fair = 2, Good = 3, Excellent = 4); Frequency of Internet health information seeking (Never = 1, several times/year = 2, several times/month = 3, several times/week = 4, several times/day = 5); Computer proficiency (Very poor = 1, Poor = 2, Fair = 3, Good = 4, Excellent = 5); Smart wearable device use (Never = 1, <3mo = 2, 3–6mo = 3, 6–12mo = 4, ≥1 yr = 5); Days feeling energetic/week (0 = 1, 1–2 = 2, 3–5 = 3, ≥5 = 4); Diversity of health information topics searched (1–2 = 1, 3–4 = 2, 5–6 = 3).

## Discussion

### Overall status of e-health literacy

The survey of 923 sport majors indicated that the mean e-health literacy score was 41.91 ± 6.90. Dimension specific scores ranked from highest to lowest as follows: critical e-health literacy (17.78 ± 3.73), interactive e-health literacy (14.52 ± 2.99), and functional e-health literacy (9.61 ± 2.57). These findings suggest that sport majors demonstrate relatively better performance in critical evaluation of online health information, with a certain capacity to discern authenticity and reliability, yet exhibit notable deficiencies in basic health information acquisition and comprehension (functional literacy), which may constrain their ability to effectively utilize electronic health resources. This “advanced competencies preceding foundational skills” imbalance aligns closely with findings reported by Li et al. (16) among university students in Taipei who also observed higher critical literacy relative to functional literacy, and remains consistent with Yoon et al.’s investigation of e-health literacy in the general South Korean population (15), which documented a similar hierarchical disparity. Taken together, these cross regional consistencies suggest that the functional critical gap is not idiosyncratic to a single educational system but may reflect a broader challenge in health education delivery across East Asian contexts. This indicates that health education in Chinese sports colleges may need to rebalance its pedagogical emphasis—potentially maintaining its strengths in fostering critical evaluation while devoting greater attention to building fundamental information retrieval, reading, and application skills, so as to promote a more comprehensively developed e-health literacy profile.

The prevalence of proficient e-health literacy among sport majors was 22.2%. Given the scarcity of prior research specifically targeting e-health literacy in this population, direct comparison with homogeneous studies was not feasible. Nevertheless, situating our findings within the broader landscape of e-health literacy research on general university students may provide a useful reference point for understanding the relative standing of sport majors, albeit with caution regarding population heterogeneity. Comparative analysis with existing research on university students revealed that this prevalence exceeded rates reported for general university students in Henan Province in 2025 (16.6%) (4), non-medical students in Wuhan in 2024 (7.91%) (3), and university students in Guangzhou in 2021 (20.3%) (5). However, it remained lower than rates documented for university students in Dali in 2025 (22.40%) (17), Dongguan in 2022 (48.8%) (18), Guangzhou in 2022 (47.74%) (19), and Nanjing in 2018 (22.97%) (6). Several factors may account for these disparities. The relatively lower rates observed in Henan and Wuhan may reflect regional differences in digital health infrastructure and health education resources, whereas the higher rates documented in Dongguan and Guangzhou are likely attributable to the advanced digital infrastructure and broader health information accessibility characteristic of the Pearl River Delta region. The near convergence of our finding with rates reported in Dali (22.40%) and Nanjing (22.97%) suggests that sport majors, despite their unique training contexts, may not inherently differ substantially from general university students in overall e-health literacy proficiency—a possibility that warrants further investigation. Additionally, temporal variations across studies (2018–2026) and differences in measurement instruments may also contribute to the observed heterogeneity. Taken together, these findings indicate that e-health literacy among sport majors currently remains at a relatively suboptimal level, yet the variability across regions and populations also highlights that sport majors—who are uniquely positioned to benefit from digital health resources due to their training related health demands—may represent a particularly responsive target group for tailored e-health interventions.

### Analysis of factors influencing e-health literacy

Nine factors emerged as significant predictors of e-health literacy among sport majors: ethnicity, gender, self-rated health status, social integration, frequency of Internet use for health information seeking, computer literacy, and diversity of health information topics accessed, among others. Detailed analysis revealed that Han ethnicity students and female students demonstrated relatively higher proficiency rates. The observed ethnic and gender disparities corroborate prior research conducted in comparable university student populations (20–23). This gender gap may be attributed to female students’ generally greater health consciousness and more proactive health information seeking behaviors, which in the sport specific context may be further amplified as health knowledge directly pertains to training and athletic performance. Notably, the observed gender disparity in this study diverges from findings reported by Shi et al. (24), a discrepancy potentially attributable to their focus on nursing students in vocational colleges, likely because their systematic health training may attenuate gender based disparities that remain pronounced among sport majors. Alternatively, the discrepancy may reflect differences in sample gender composition: our sample was predominantly male (78.2%), whereas Shi et al. studied primarily female nursing students. This imbalance may have amplified the gender difference in our study while attenuating it in theirs, consistent with evidence that e-health literacy related associations are stronger in males than females (25). The ethnic disparity may reflect linguistic barriers in accessing Chinese dominant digital health resources, compounded by regional educational disadvantages disproportionately affecting minority students from less developed western provinces. These results suggest that targeted interventions should prioritize ethnic minority and male students, with culturally and gender sensitive strategies addressing their specific barriers to digital health information access.

Additional findings indicated that students with better self-rated health status, harmonious interpersonal relationships, morning energy levels exceeding 5 days per week, and daily consumption of fish, poultry, meat, and eggs exhibited superior e-health literacy levels—consistent with previous research (26, 27). Several plausible explanations may account for these associations. Students with better self-rated health are likely to have greater health awareness and stronger motivation to seek health information, potentially creating a positive feedback loop between health status and health literacy. Harmonious interpersonal relationships may also play a role by facilitating health information exchange within social networks, thus broadening individuals’ exposure to diverse health knowledge. Regarding lifestyle factors, higher energy levels and balanced dietary intake could reflect broader health-conscious patterns, which often co-occur with proactive health information engagement. Of particular significance, information behavioral factors (frequency of health information seeking, diversity of search content), computer proficiency, and use of smart wearable devices (e.g., fitness trackers) demonstrated among the largest effect sizes. These findings highlight that digital skills and active information practice, rather than passive information reception, are key drivers of e-health literacy. The strong effect of smart wearable device use suggests that interactive health technologies may foster sustained user engagement with personal health data, thereby reinforcing health literacy development through experiential learning. Taken together, these findings underscore that improving digital skills and information practice capabilities represents a critical pathway for enhancing e-health literacy in this population, whereas the role of emerging digital health tools—particularly wearables that provide real time health feedback—warrants further investigation in future research.

This study reveals that e-health literacy among sport majors is associated with the synergistic interplay of multiple factors, with information behavior and digital skills emerging as particularly prominent correlates. These findings provide empirical evidence for physical education institutions to precisely target health information literacy education and optimize curriculum development. Future interventions should prioritize strengthening practical training in Internet health information retrieval, broadening the scope of health knowledge exposure, and addressing the differentiated needs of male students, and fostering interpersonal and social factors, particularly interpersonal harmony, which may facilitate health information sharing through social networks. Such multi-faceted approaches would comprehensively advancing the digital health competency of physical education professionals.

### Limitations and future directions

This study employed a cross sectional design, precluding the establishment of causal relationships among variables. Additionally, the sample was confined to sports colleges within six universities in Jiangsu Province, necessitating caution in generalizing findings to the national context. Moreover, the directional relationship between e-health literacy and certain health behavior indicators (e.g., dietary habits, sleep patterns) remains ambiguous, with potential bidirectional relationships or confounding by third variables. Furthermore, the predominantly male sample may have amplified the observed gender differences. Future research should adopt longitudinal designs and integrate qualitative methodologies to elucidate the formation mechanisms of health information behaviors among physical education students. Additionally, studies with more gender-balanced samples are needed to verify whether the observed gender-related findings hold across different sex compositions.

## Data Availability

The original contributions presented in the study are included in the article/supplementary material, further inquiries can be directed to the corresponding authors.

## References

[1] Liu Z Zhang H Zhang Y Du CC Li HJ Zhao J . Analysis of e-health literacy status and influencing factors among rural elderly in Zhengzhou City. Mod Prev Med. (2020) 47:283–6.

[2] Sørensen K Van den Broucke S Fullam J Doyle G Pelikan J Slonska Z . Health literacy and public health: a systematic review and integration of definitions and models. BMC Public Health. (2012) 12:80. doi: 10.1186/1471-2458-12-80, 22276600 PMC3292515

[3] Yu RR Liao WJ Che L Fang Y Zheng TY. Analysis of e-health literacy status and influencing factors among non-medical university students in Wuhan City. Occup Health. (2024) 40:375–80. doi: 10.13329/j.cnki.zyyjk.2024.0054

[4] Wu JL Zhang Y Rong YR Wu L Xi ST Wu KM . Study on e-health literacy status and influencing factors among university students in 4 universities in Henan Province. Zhongguo Jian Kang Jiao Yu. (2025) 41:244–51. doi: 10.16168/j.cnki.issn.1002-9982.2025.03.009

[5] Mai JR Zhou L Lin LN. A cross-sectional study on e-health literacy among university students in Guangzhou. Health Vocat Educ. (2021) 39:56–7.

[6] Meng SX Shen C. Investigation on e-health literacy and behavior status among university students in a Nanjing university. Chin J Health Educ. (2018) 34:254–7. doi: 10.16168/j.cnki.issn.1002-9982.2018.03.014

[7] Ishikawa H Miyawaki R Kato M Muilenburg JL Tomar YA Kawamura Y. Digital health literacy and trust in health information sources: a comparative study of university students in Japan, the United States, and India. SSM Popul Health. (2025) 31:101844. doi: 10.1016/j.ssmph.2025.101844, 40746760 PMC12311546

[8] Geiger S Esser AJ Marsall M Muehlbauer T Skoda EM Teufel M . Association between eHealth literacy and health outcomes in German athletes using the GR-eHEALS questionnaire: a validation and outcome study. BMC Sports Sci Med Rehabil. (2024) 16:117. doi: 10.1186/s13102-024-00902-9, 38790069 PMC11127337

[9] Zhou YZ Wang Q. The era mission and responsibility of college students majoring in physical education in the construction of healthy China. Shandong Sports Sci Technol. (2024) 46:66–71. doi: 10.14105/j.cnki.1009-9840.2024.02.005

[10] Yin Y Fang Q Min Z Liu T. Health literacy level and its influencing factors among college students majoring in sports: a cross-sectional study. BMC Public Health. (2025) 25:3690. doi: 10.1186/s12889-025-24998-x, 41174695 PMC12576981

[11] Liu HX Chow BC Hu C Hassel H Huang WY. EHealth usage among Chinese college students: qualitative findings. BMC Public Health. (2022) 22:1088. doi: 10.1186/s12889-022-13521-1, 35642028 PMC9158198

[12] Chiang CH Yang SQ Xu WZ. Construction of college students' online health literacy scale and its relationship with health behaviors. Formosa J Mental Health. (2015) 28:389–420.

[13] Yang SC Luo YF Chiang CH. The associations among individual factors, eHealth literacy, and health-promoting lifestyles among college students. J Med Internet Res. (2017) 19:e15. doi: 10.2196/jmir.5964, 28073739 PMC5263862

[14] Zhang XH. A Mixed-Methods Study on e-Health Literacy status of Vocational Medical Students. Nanjing: Nanjing Medical University (2020).

[15] Yoon J Cho J Kang S Oh K Choi S Kang Y. Development of health literacy index for the Korea National Health and nutrition examination survey. Public Health Wkly Rep. (2023) 16:709–25. doi: 10.56786/PHWR.2023.16.23.1, 41334091 PMC12480063

[16] Li Q Fang F Zhang Y Tu J Zhu P Xi L. Ehealth literacy and its outcomes among post secondary students: systematic review. J Med Internet Res. (2025) 27:e64489. doi: 10.2196/64489, 40601925 PMC12278882

[17] Fan ZY Chen HY. Analysis of e-health literacy status and influencing factors among university students in Dali. J Dali Univ. (2025) 10:75–81.

[18] Jiang LH Guo XY Lu BY Deng GJ Wu ZH Lyu GA. Correlation between e-health literacy and physical health among university students. Chin J Sch Health. (2022) 43:990–4. doi: 10.16835/j.cnki.1000-9817.2022.07.008

[19] Zou XJ. Investigation on e-health literacy status among university students in Guangzhou. Health Educ Health Promot. (2022) 17:577–80. doi: 10.16117/j.cnki.31-1974/r.202206577

[20] Zhang LN Han CW Pan WB. Research status and progress of health literacy among domestic university students. Occup Health. (2020) 36:573–6. doi: 10.13329/j.cnki.zyyjk.2020.0151

[21] Zhang JJ Zhong M Chen ZX. Study on the association between e-health literacy and health behaviors among university students in Guangzhou. Zhongguo Gong Gong Wei Sheng Guan Li. (2023) 39:497–501. doi: 10.19568/j.cnki.23-1318.2023.04.0015

[22] Zhang P Zhang XF Yuan H Hou YW An XF Hao YH . Investigation on communication effect of new media health information based on public perspective and analysis of influencing factors. Zhongguo Gong Gong Wei Sheng Guan Li. (2023) 39:194–7.

[23] Tsukahara S Yamaguchi S Igarashi F Uruma R Ikuina N Iwakura K . Association of eHealth literacy with lifestyle behaviors in university students: questionnaire-based cross-sectional study. J Med Internet Res. (2020) 22:e16216. doi: 10.2196/18155 PMC7381004 32579126

[24] Shi XY. Analysis of e-health literacy status and influencing factors among nursing students. Contin Med Educ. (2025) 39:158–61.

[25] Fu Y Lv D Chen R Feng K Li B Li Q . Association between social capital, eHealth literacy, and digital health literacy among undergraduate students in China: a multigroup analysis based on gender differences. Front Public Health. (2026) 14:1839874. doi: 10.3389/fpubh.2026.1839874, 42267284 PMC13243295

[26] Li SJ Yin YT Chen LJ Zhang P Cui GH. Analysis of e-health literacy level and influencing factors among university students in Jinan City. Chin J Sch Health. (2019) 40:1071–4. doi: 10.16835/j.cnki.1000-9817.2019.07.031

[27] Liu JC Yin YT Fan YY. Relationship between e-health literacy and illness behavior among vocational college students in Jinan City. Chin J Sch Health. (2020) 41:1502–5. doi: 10.16835/j.cnki.1000-9817.2020.10.016

